# Two-Terminal Nonvolatile Write-Once-Read-Many-Times Memory Based on All-Inorganic Halide Perovskite

**DOI:** 10.3390/mi14010093

**Published:** 2022-12-29

**Authors:** Zhipeng Yu, Xiaofeng Zhao, Chunpeng Ai, Xin Fang, Xiaohan Zhao, Yanchao Wang, Hongquan Zhang

**Affiliations:** Heilongjiang Provincial Key Laboratory of Micro-Nano Sensitive Devices and Systems, Heilongjiang University, Harbin 150080, China

**Keywords:** write-once-read-many-times (WORM), memory, all-inorganic halide perovskite

## Abstract

Write-once-read-many-times (WORM) memory belonging to an important non-volatile memory type achieves the read-only state after the write operation and is used in the fields of data security storage widely. WORM memory has been developed based on a variety of materials. In recent years, halide perovskites have become the research hotspot material for this memory due to its excellent properties. Here, the all-inorganic CsPbBr_3_ perovskite thin film was prepared on a FTO substrate by using a two-step method. The prepared CsPbBr_3_ thin films have the characteristics of densely packed crystal grains and smooth surface. The device, having the FTO/CsPbBr_3_/Al sandwich structure by evaporating the Al electrode onto the CsPbBr_3_ thin film, represents the typical WORM behavior, with long data retention time (10^4^ s), a low operation voltage (2.1 V) and a low reading voltage (0.1 V). Additionally, the resistance transition mechanism of the resulting WORM devices was analyzed.

## 1. Introduction

The technological advancement of Big Data, artificial intelligence and integrated circuit has prompted the rapid development of modern information technology [1]. The memory devices with high efficiency and reliability are indispensable in order to adapt to the development of modern information technology [2,3]. There are many different types of memory devices using similar device structures. In previous work, devices based on ITO/PFOxPy/Al structures with p-type PFOxPy as the active medium showed Dynamic Random Access Memory (DRAM) characteristics, attributed to the memory switch mechanism of the Schottky barrier and shallow traps [4]. Ge et al. proposed the Resistive Switching Random-Access Memory (ReRAM) with ultralow bias, based on the FTO/CsBi_3_I_10_/Ag and FTO/Cs_3_Bi_2_I_9_/Ag structure, where the resistive behavior is attributed to conductive filaments formed by halide vacancies [5]. Recently, Guan et al. reported the Photonic Resistive Switching Memory with an Ag/SrTiO_3_/CsPbBr_3_/Au heterogeneous structure, with operational mechanisms of photonic heterojunction barrier variation and conductive filaments formed by metal electrodes [6]. Hsu et al. fabricated Write-Once-Read-Many-Times Memory (WORM) based on the Al/AlO_x_:N/n^+^-Si structures and analyzed the operational mechanism of the limited carrier transport by the defects and P-F emission [7]. Among such devices, the WORM devices have been widely used in various permanent storage applications [8]. In the future development of WORM, it is possible that the data only needs to set up the read-only state and not the writing permission, meeting the needs for safe storage enterprises.

To date, WORM characteristics have been observed in various materials, including organics [9,10], metal oxides [11,12,13,14], biological materials [15], low-dimension materials [16] and halide perovskites [17]. Furthermore, increasing attention has been paid to the halide perovskites due to their excellent properties, including simple preparation process of the precursors from solution [18], high defect tolerance [19], flexibility [20] and ion conductivity [21]. The general chemical formula for perovskite is AMX_3_, in which A is the monovalent cation, M is the divalent cation and X is the monovalent anion [22]. In AMX_3_ structure, the corner-sharing MX_6_ octahedra form an extended three-dimensional network, with A cations residing in the cuboctahedron spaces formed by the M-X framework [23]. Usually, the large A cations are CH_3_NH^3+^, CH(NH_2_)^2+^ and Cs^+^, the small M cation is Pb^2+^, and the X anions are the halogen ions (such as Cl^−^, Br^−^, I^−^) [24]. In the past, significant research has focused on memory based on the organic–inorganic hybrid halide perovskites (OHP), due to its excellent performance [25,26]. However, due to the hygroscopicity and volatility of organic cations, the OHP may be chemically unstable under the condition of oxygen, moisture and high temperature, limiting its application [27]. To form a more stable perovskite structure, many researchers have attempted to replace the organic cation of the A-site with inorganic Cs^+^ [28].

Here, we synthesized a CsPbBr_3_ perovskite structure using the solution method and fabricated a FTO/CsPbBr_3_/Al sandwich structure device using an all-inorganic perovskite CsPbBr_3_ thin film as an active layer, representing the WORM memory characteristics. Additionally, the typical WORM behaviors of the device were investigated.

## 2. Materials and Methods

### 2.1. Chemicals and Reagents

Cesium bromide (CsBr, 99.999%), lead bromide (PbBr_2_, 99.99%) and dimethyl formamide (DMF, >99.9%) were purchased from Aladdin Holdings Group Co., Ltd (Hong Kong, China). Methyl alcohol (99.99%) was purchased from Shanghai Macklin Biochemical Technology., Ltd (Shanghai, China). FTO conductive substrate was purchased from Luoyang Guluo Glass Co., Ltd (Henan, China). All materials were used directly without further purification.

### 2.2. Test Equipment

The phase analysis of the prepared thin film was carried out by X-ray diffraction (XRD, Cu Kα radiation with λ = 1.5418 Å, Bruker D8 ADVANCE, Karlsruhe, Germany). The surface morphology of the prepared thin film was observed by a scanning electron microscope (SEM, TESCAN AMBER, Brno, Czech Republic) and atomic force microscope (AFM, Bruker Multimode8, Billerica, MA, USA). The cross-sectional image of prepared thin films was observed by a scanning electron microscope (SEM, Hitachi S3400, Tokyo, Japan). The memory current-voltage scanning was performed using a semiconductor parameter analyzer (Keithley 4200-SCS, Tektronix, Beaverton, OR, USA).

### 2.3. Fabrication of the Memory Device

**Figure 1:**
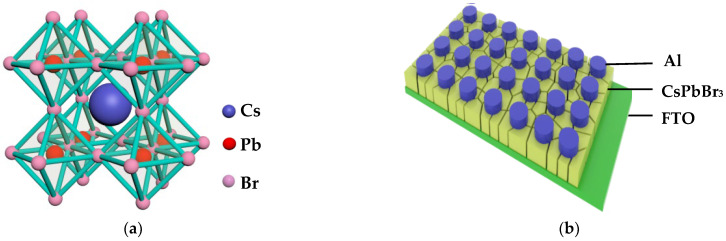
The crystal structures of CsPbBr_3_ and the schematic drawing of FTO/CsPbBr_3_/Al vertical stack structure for the memory. (**a**) The crystal structures of CsPbBr_3_; (**b**) the schematic drawing of FTO/CsPbBr_3_/Al vertical stack structure for the memory.

FTO substrates were sequentially cleaned for 20 min with acetone, isopropanol, ethyl alcohol and deionized water and then dried under nitrogen flow. The PbBr_2_ was dispersed in DMF with magnetic stirring for 7 h at 70 °C to prepare the 1.0 M PbBr_2_ precursor solution. The CsBr was added in methyl alcohol with magnetic stirring for 7 h at 50 °C to obtain the 0.07 M CsBr precursor solution. Firstly, the PbBr_2_ precursor solution was spin-coated onto the FTO substrate with 2000 rpm for 30 s; then, the substrate was dried at 75 °C for 30 min to form PbBr_2_ thin film. Secondly, the PbBr_2_ thin film was dipped in the CsBr solution at 50 °C for 10 min. Thereafter, the substrate was annealed at 160 °C for 30 min to accomplish perovskite thin film preparation. Finally, Al top electrodes with the diameter of 200 μm were deposited on the CsPbBr_3_ thin film by vacuum evaporation through shadow mask. Figure 1 shows the crystal structures of CsPbBr_3_ [29] and the schematic drawing of the FTO/CsPbBr_3_/Al vertical stack structure for the memory.

## 3. Results

### 3.1. Material Characterization

**Figure 2:**
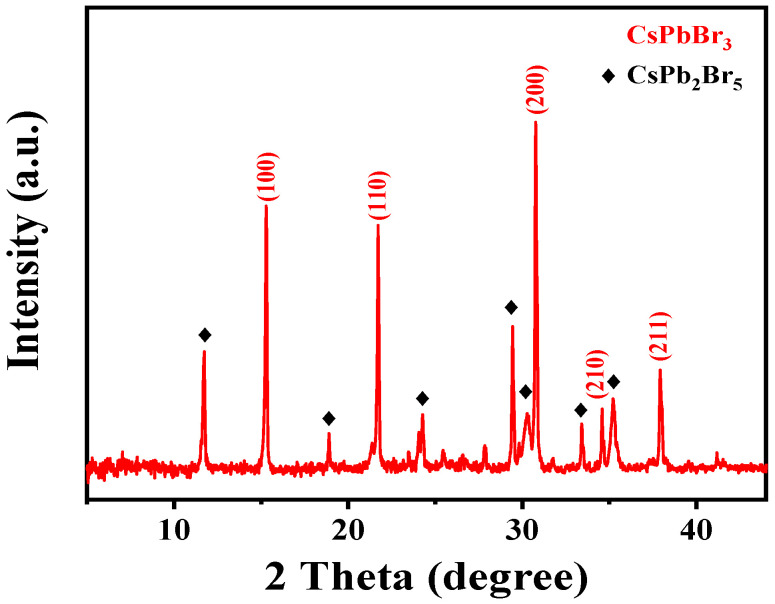
The X-ray diffraction spectra of the synthesized thin film.

Figure 2 depicts the XRD measurements spectrum of the synthesized thin film to analyze the phase composition and crystallographic information. The prominent and intensely sharp three peaks indexed as (100), (110) and (200) at 15.18°, 21.55° and 30.64° confirmed the higher crystallinity of CsPbBr_3_ (PDF#54-0752). Some additional weak diffraction peaks with a black rhombus correspond to another phase of perovskite as represented by CsPb_2_Br_5_ (PDF#25-0211). The CsPb_2_Br_5_ with a two-dimensional-layer perovskite structure possibly grew as an intermediate product during the preparation of CsPbBr_3_ thin film by the low temperature synthesis process [2]. The small area and weak diffraction peaks of the XRD spectrum demonstrate the presence of a small amount of CsPb_2_Br_5_. Moreover, it was reported that the CsPb_2_Br_5_ possesses similar behavior to CsPbBr_3_ [2]. Therefore, a small amount of CsPb_2_Br_5_ exhibits little influence on memory behaviors [20].

**Figure 3:**
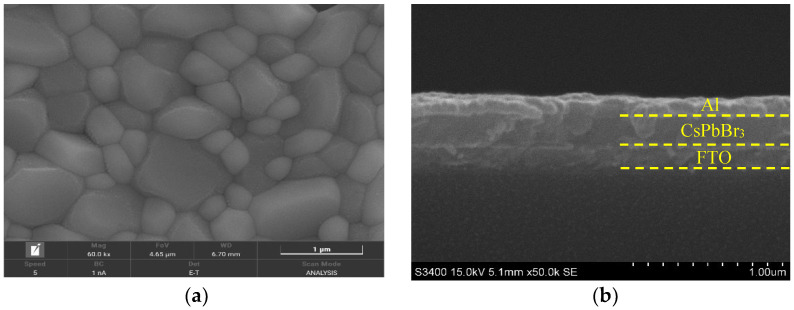
The images of SEM for CsPbBr_3_ thin film. (**a**) The surface SEM image; (**b**) the cross-sectional SEM image.

**Figure 4:**
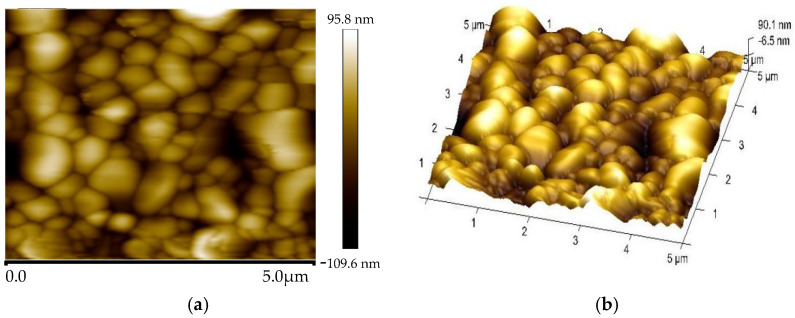
The AFM images of the CsPbBr_3_ thin film. (**a**) AFM image; (**b**) corresponding 3D height image.

The surface morphology of the CsPbBr_3_ thin film was investigated by SEM and AFM. Figure 3a shows the surface SEM image, in which densely packed crystal grains and pinhole-free surface can be observed. Figure 3b gives the cross-sectional SEM image of the device, visualizing the CsPbBr_3_ layer and the Al electrode thickness of ~230 nm and ~100 nm, respectively. Figure 4 is the AFM image and the corresponding 3D AFM height image (scan size = 5 μm × 5 μm), where the CsPbBr_3_ thin film exhibits a relatively smooth surface.

### 3.2. Current-Voltage Characteristic

**Figure 5:**
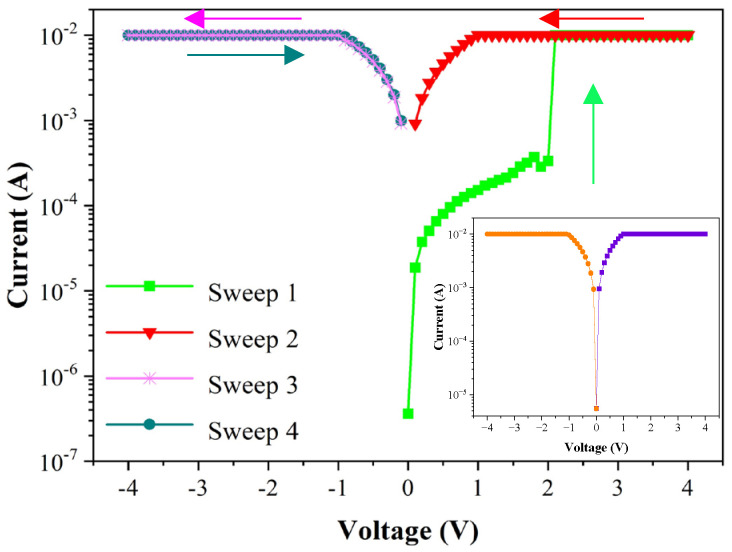
The *I*-*V* characteristic curves of the FTO/CsPbBr_3_/Al memory device. The illustration demonstrates that the device is always kept at a low resistance state during the subsequent scanning voltage.

**Figure 6:**
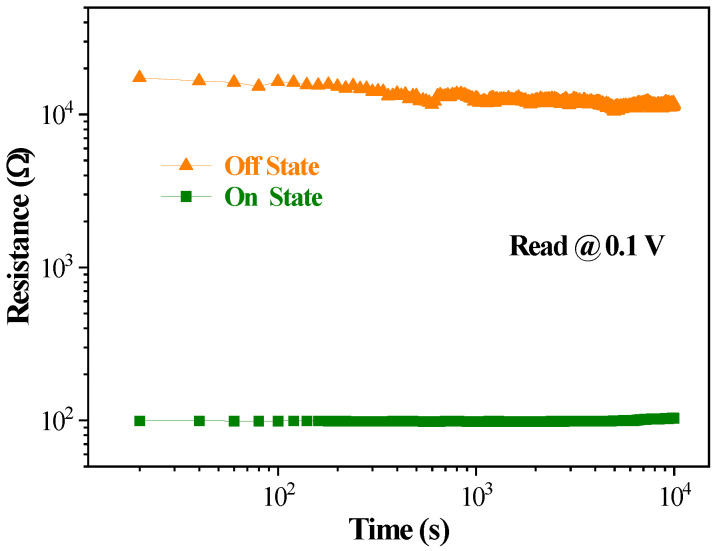
The retention characteristics the of FTO/CsPbBr_3_/Al memory device.

The current-voltage (*I*-*V*) characteristics were measured using Keithley 4200-SCS, where the Al electrode was connected to the positive pole and the FTO was connected to the ground. Figure 5 shows the *I*-*V* characteristic curves of the FTO/CsPbBr_3_/Al device when exerting the sweep voltage in the sequence of 0→+4→0→−4→0. During the test, the device was set at a compliance current of 10^−2^ A to prevent the device from being damaged by excessive current. The pristine device was at the high resistance state (HRS), corresponding to the OFF state. When exerting the sweeping voltage from 0 V to 4 V (sweep 1), the current was enhanced with the increasing applied voltage, progressively. The rapidly increasing current at 2.1 V indicates that the device completed the writing operation in which the resistance transformed from an HRS to a low resistance state (LRS), corresponding to the ON state. After that, the device remained in the ON state in the subsequent voltage sweeps, i.e., sweep 2 (+4→0 V), sweep 3 (0→−4 V) and sweep 4 (−4→0 V). The *I*-*V* characteristics of the device demonstrate that once the data were written, it could only be read and could not be rewritten. The retention characteristics of the device are represented in Figure 6. The HRS and LRS of the device had no obvious variations for 10^4^ s at a read voltage of 0.1 V. The results indicate the device possesses excellent operation stability. Many the WORM devices have been reported based on various materials by other groups, as seen in Table 1, which shows a brief summary of the WORM performances based on our research and that of the current literature. Comparing the WORM performance, it can be seen that the device in this study has excellent retention performance, low operation voltage and small reading voltage.

## 4. Discussion

**Figure 7:**
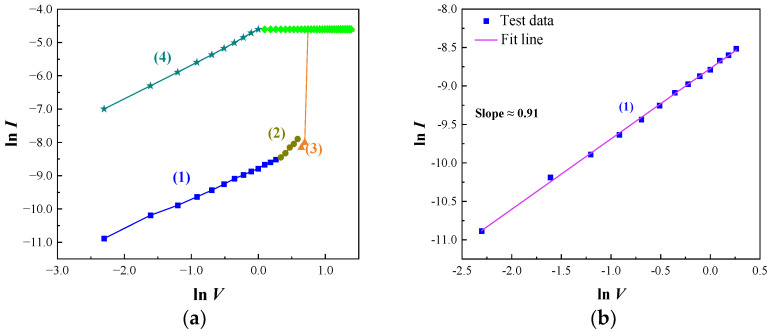

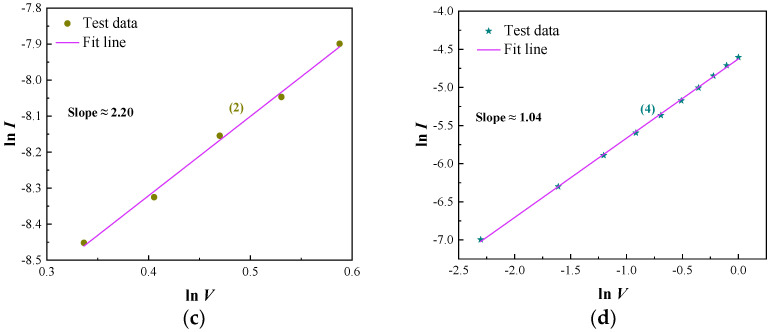
The measured and fitted *I*-*V* characteristic curves of the FTO/CsPbBr_3_/Al device. (**a**) Double logarithmic *I*-*V* curve of forward scanning voltage. (**b**) Measured and fitted line of *I*-*V* characteristic in 0–1.3 V. (**c**) Measured and fitted line of *I*-*V* characteristic in 1.4–1.8 V. (**d**) Measured and fitted line of *I*-*V* characteristic at a LRS.

In general, there are two possible mechanisms to explain the resistive switching effect: the formation and rupture of conductive filaments in the active medium [34] and the interface barrier variation between the active medium and the adjacent layers [35]. To investigate the resistance transition mechanism of the resulting WORM devices, the *I*-*V* characteristic curves in the forward sweeping voltage regions are converted to the double-logarithmic coordinate scales, as shown in Figure 7. In Figure 7a, the *I*-*V* curve was divided into four distinct regions for analysis. In Figure 7b, the slope of the fitting curve is approximate to 1 in the process (1) (0–1.3 V), indicating an Ohmic dominant conduction (*I*∝*V*) [36,37]. It is inevitable that randomly distributed defects are formed in the CsPbBr_3_ thin film during the process of low-temperature solution synthesis [38], resulting in some unfilled trap energy levels. In the CsPbBr_3_ perovskite crystal, Br^−^ ions (Br^−^ vacancies) have the smallest activation energy, which makes it possible to primarily migrate species under the electric field [39]. Under a low applied voltage, Br^−^ ions migrate toward the anode and leave behind Br^−^ vacancies. The density of thermally generating free carriers within the thin film is predominant over the injected charge carriers [40]. A small number of carriers injected into CsPbBr_3_ are captured by the vacancy energy levels, contributing minorly to the current. In the device, the current is mainly caused by the thermally exciting intrinsic carrier drift. Therefore, the current has a linear relationship with the applied voltage, showing ohmic conductivity. In Figure 7c, the slope of the fitting curve approximates to 2 in the process (2) (1.4–1.8 V). With the increasing applied voltage, the number of injected carriers increases; meanwhile, the vacancies captured energy level is gradually filled by the injected carriers. When the injected carriers dominate the current, the *I*-*V* relationship confirms to Mott–Gurney law [41]. In this process, the current has a linear relationship with the square of voltage, as shown in following equation [42]:

(1) $$J=\frac{9}{8}\theta_{0}\varepsilon_{0}\varepsilon_{r}\mu \frac{V^{2}}{L^{3}}$$

where *J* is the current density of the device, *μ* is electronic mobility, *V* is the voltage applied to the device, *θ*_0_ is the ratio of free electrons relative to trapped electrons, *ε*_0_ and *ε*_r_ are the vacuum dielectric constant and the relative dielectric constant of the active layer, respectively, and *L* is thickness of the active layer. In the HRS region, the conductive mechanism is consistent with the typical trap-controlled space charge limited currents (SCLC) [6]. In the transition region from process (2) to (3), a negative differential resistance shows that the abnormal behavior of the current decreases with the increasing bias voltage, as shown in Figure 7a. The holes injected from the anode begin transition through the thin film at the threshold voltage, causing some holes traps to be filled. At this time, the life of the hole will increase with the reduction of that of the electron, resulting in a negative differential resistance [43,44]. Thereafter, rapidly increasing current to the limiting current indicates the device is up to an LRS due to the formation of a conductive channel connected by the Br^−^ vacancies between the two electrodes. In Figure 7d, the slope of the fitting curve approximates to 1 in the flyback voltage region in the process (4) (1–0 V), showing ohmic conductivity. Meanwhile, the formed robust conductive filaments composed of Br^−^ vacancies make it possible for the device to remain at an LRS. When exerting a negative sweeping voltage, the device remains in an LRS. Therefore, it is possible that the device can exhibit the WORM characteristics after the writing process.

## 5. Conclusions

In this research, we fabricated a WORM device based on the resulting CsPbBr_3_ thin film. The CsPbBr_3_ thin film was synthesized by a two-step method. The FTO/CsPbBr_3_/Al device demonstrated the typical WORM characteristics, including low operation voltage, small reading voltage and long data retention time. Through the linear fitting of the experimental data to construct a conducting filament model, the memory behavior conforms to the Ohmic mechanism and SCLC mechanism.

## Figures and Tables

**Table 1:** A brief summary of the WORM performance based on our research and other studies.

Device Structure	Memory Type	Operation Voltage (v)	Reading Voltage (v)	Retention Time (s)	Ref.
Al/Au NPs: lignin/Al	WORM	4.7	0.6	>10^3^	[30]
ITO/CsPbBr_3_ QDs/Au	WORM	−1.1	−0.5	>10^3^	[8]
Au/ZnO MBs/Au	WORM	≈6.5	0.35	≈10^4^	[31]
n^+^-Si/CuO/Ag	WORM	≈3	1	10^5^	[32]
Ag/WS_2_-PVP/Cu	WORM	<1	0.02	>2 × 10^2^	[33]
FTO/CsPbBr_3_/Al	WORM	2.1	0.1	10^4^	This work

## Data Availability

Not applicable.
